# Crystal structures of the triple perovskites Ba_2_K_2_Te_2_O_9_ and Ba_2_KNaTe_2_O_9_, and redetermination of the double perovskite Ba_2_CaTeO_6_

**DOI:** 10.1107/S2056989018009064

**Published:** 2018-06-26

**Authors:** Matthias Weil

**Affiliations:** a Institute for Chemical Technologies and Analytics, Division of Structural Chemistry, TU Wien, Getreidemarkt 9/164-SC, A-1060 Vienna, Austria

**Keywords:** crystal structure, perovskite family, 6*H*-BaTiO_3_ structure type, double perovskite, isotypism

## Abstract

Single crystals of Ba_2_K_2_Te_2_O_9_ (dibarium dipotassium nona­oxidoditellurate), (I), Ba_2_KNaTe_2_O_9_ (dibarium potassium sodium nona­oxidoditellurate), (II), and Ba_2_CaTeO_6_ (dibarium calcium hexa­oxidotellurate), (III), were obtained from KNO_3_/KI or KNO_3_/NaNO_3_ flux syntheses in platinum crucibles for (I) and (II), or porcelain crucibles for (III). (I) and (II) are isotypic and are members of triple perovskites with general formula *A*_2_^[12*co*]^ *A*′^[12*co*]^ *B*_2_^[6*o*]^ *B*′^[6*o*]^O_9_. They crystallize in the 6*H*-BaTiO_3_ structure family in space-group type *P*6_3_/*mmc*, with the *A*, *A*′, *B* and *B*′ sites being occupied by K, Ba, Te and a second Ba in (I), and in (II) by mixed-occupied (Ba/K), Ba, Te and Na sites, respectively. (III) adopts the *A*_2_^[12*co*]^ *B′* ^[6*o*]^ *B′′* ^[6*o*]^O_6_ double perovskite structure in space-group type *Fm*  *m*, with Ba, Ca and Te located on the *A*, *B*′ and *B′′* sites, respectively. The current refinement of (III) is based on single-crystal X-ray data. It confirms the previous refinement from X-ray powder diffraction data [Fu *et al.* (2008). *J. Solid State Chem.* **181**, 2523–2529], but with higher precision.

## Abstract 2

The triple perovskites Ba_2_K_2_Te_2_O_9_ and Ba_2_KNaTe_2_O_9_ crystallize in the 6*H*-BaTiO_3_-type of structure, whereas Ba_2_CaTeO_6_ represents a double perovskite (cubic elpasolite structure type).

## Chemical context   

During a recent project on the structure determination of barium oxotellurates(VI), different preparation methods were applied for single-crystal growth of the phases Ba[H_4_TeO_6_], Ba[H_2_TeO_5_], Ba[Te_2_O_6_(OH)_2_] and Ba[TeO_4_] (Weil *et al.*, 2016 ▸). Owing to the different water content that defines the thermal stability range of the respective phase, relatively mild temperatures < 600 K had to be adjusted for the three hydrous phases using either a diffusion method in aqueous solutions (room temperature) or hydro­thermal methods (*ca* 470 K), whereas for the anhydrous phase higher temperatures could be employed. However, Ba[TeO_4_] decomposes into Ba[TeO_3_] with release of oxygen at temperatures above 1000 K, which prevents prolonged heating near this temperature. Although very small crystals of Ba[TeO_4_] with a rather poor quality could eventually be grown by heating Ba[H_4_TeO_6_] at 873 K for some days (Weil *et al.*, 2016 ▸), alternative crystal-growth methods were tested with the intention of obtaining larger crystals with better quality. With the upper stability range of the target phase Ba[TeO_4_] in mind, KNO_3_/KI or KNO_3_/NaNO_3_ mixtures were used for crystal-growth experiments. Such salt mixtures have low eutectic melting points, *e.g*. 498 K for a 50:50 mol% mixture of NaNO_3_/KNO_3_ (Berg & Kerridge, 2004 ▸). At least for the latter eutectic mixture, crystal-growth experiments from the melt have already been applied successfully for another barium phase, *viz.* Ba_2_As_2_O_7_ (Weil, 2016 ▸). However, Ba[TeO_4_] did not form under the given conditions because K^+^ or mixtures of K^+^ and Na^+^ were incorporated instead, resulting in the formation of Ba_2_K_2_Te_2_O_9_ (I) or Ba_2_KNaTe_2_O_9_ (II) single crystals. In the case a porcelain crucible was employed, Ba_2_CaTeO_6_ (III) was obtained in form of very few single crystals.

## Structural commentary   

The three title compounds belong to the vast family of perovskites (Tilley, 2016 ▸). The ideal cubic *A* ^[12*co*]^ *B* ^[6*o*]^O_3_ perovskite structure comprises of corner-sharing [*B*O_6_] octa­hedra. In the centre of the resulting ^3^_∞_[*B*O_6/2_] network, the *A*-site cation occupies a 12-coordinate cubocta­hedral site. The 2*H* hexa­gonal perovskite structure contains chains of face-sharing [*B*O_6_] octa­hedra that are separated by chains of *A*-site cations. In an alternative description, perovskite structures can be derived from closed-packed arrangements of the anions with different stacking sequences (Lufaso & zur Loye, 2005*a* ▸; Stöger *et al.*, 2010 ▸). For example, in the cubic perovskite an *ABC* stacking and in the hexa­gonal 2*H* perovskite an *AB* stacking is observed. More complex structures that are realized in double perovskites or triple perovskites can include both cubic (*c*) and hexa­gonal stacking sequences (*h*) and consequently structure motifs of corner-sharing and face-sharing [*B*O_6_] octa­hedra like in the triple perovskites discussed below.

**Figure 1:**
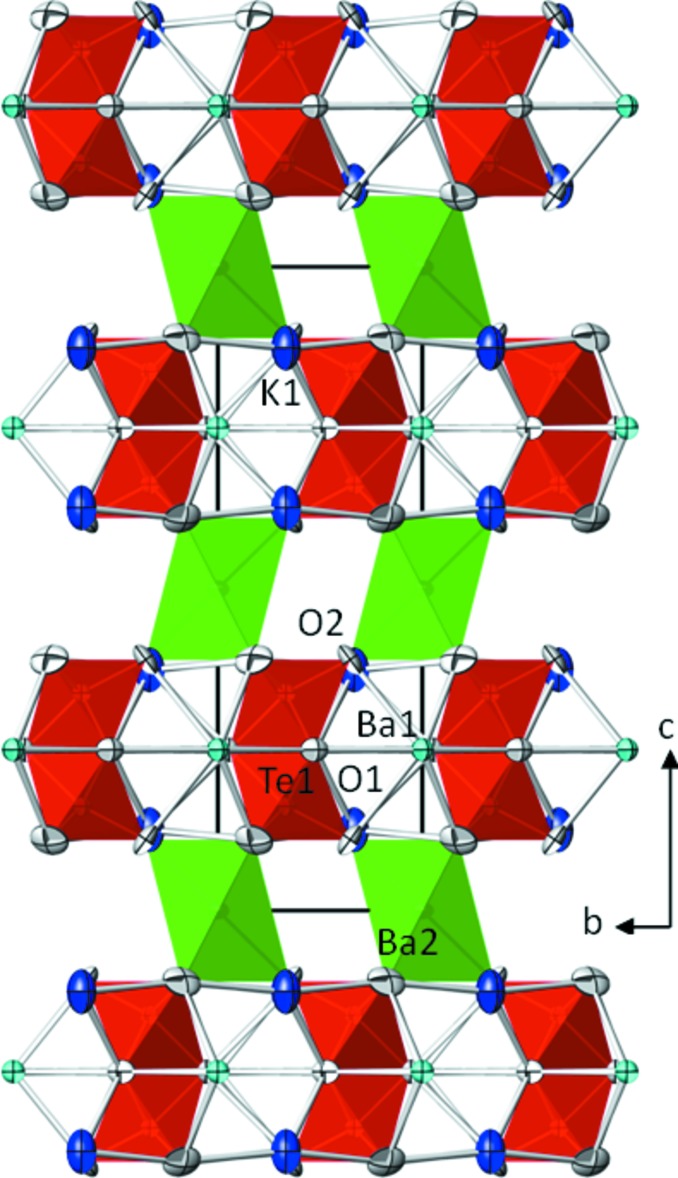
Projection of the crystal structure of Ba_2_K_2_Te_2_O_9_ (I) along [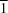
00]. [Ba2O_6_] octa­hedra are green, [TeO_6_] octa­hedra are red, potassium sites are blue, Ba1 sites turquoise and O sites shaded pale grey. Displacement ellipsoids are drawn at the 97% probability level.

Ba_2_K_2_Te_2_O_9_ (I) and Ba_2_KNaTe_2_O_9_ (II) are isotypic and members of the triple perovskite family with general formula *A*_2_^[12*co*]^ *A*′^[12*co*]^ *B*_2_^[6*o*]^ *B*′^[6*o*]^O_9_. They crystallize in the 6*H*-BaTiO_3_ structure type in space-group type *P*6_3_ */mmc* with *Z* = 2. In (I) the *A*, *A*′, *B* and *B*′ sites are occupied by K1, Ba1, Te1 and Ba2, and in (II) by mixed-occupied (Ba/K)1, Ba1, Te1 and Na2, respectively. The 6*H*-BaTiO_3_ structure type is sometimes also referred to as the BaFeO_2+*x*_ structure type with possible values for *Z* = 2, 3 or 6, dependent on the overall formula sum of the compound. The stacking sequence for this structure type is (*cch*)_2_ (Tilley, 2016 ▸). About 240 entries of this structure family are compiled in the recent version of the Inorganic Crystal Structure Database (ICSD, 2018 ▸), with hexa­gonal BaTiO_3_ being the first phase that has been structurally determined (Burbank & Evans, 1948 ▸). Only four Te-containing phases have been reported so far to adopt this structure type, *viz*. Ba_3_Fe_2_TeO_9_ (Harari *et al.*, 1972 ▸), K_3_LaTe_2_O_9_ (Zhang *et al.*, 2015 ▸), Ba_3_Cr_1.94_Te_1.06_O_9_ (Li *et al.*, 2016 ▸) and the high-pressure phase Ba_2_NiTeO_6_ (*Z* = 3; Aoba *et al.*, 2016 ▸). A review of this structure type and of perovskites in general was given recently by Tilley (2016 ▸). In both structures (I) and (II), Ba1 is situated on Wyckoff position 2*b* (site symmetry 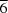 *m*2), the K1 site in (I) and the mixed-occpied (Ba/K)1 site (occupancy ratio 1:1) in (II) on 4*f* (3*m*.), Ba2 in (I) and Na2 in (II) on 2*a* (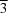 *m*.), and in both structures Te1 4*f* (3*m*.), O1 on 6*h* (*mm*2) and O2 on 12*k* (.*m*.), respectively. Hence the smaller Te^VI^ atoms occupy the face-sharing octa­hedral *B* site while the larger barium (Ba2 in (I)) or sodium cations (Na2 in (II)) occupy the corner-sharing octa­hedral *B*′ site. The inner angles of the two face-sharing [TeO_6_] octa­hedra in (I) and (II) (Table 1 ▸) are more similar than those in isotypic triple perovskites (Lufaso & zur Loye, 2005*a* ▸), with center shifts of 0.076 Å in (I) and of 0.191 Å in (II). Representative for both (I) and (II), the crystal structure of Ba_2_K_2_Te_2_O_9_ is given in Fig. 1 ▸. It should be noted that the *A* (= K1) position in (I) has only nine coordination partners, while in (II) twelve oxygen atoms surround the corresponding site that is statistically occupied by Ba^2+^ and K^+^ (= (Ba/K)1).

**Figure 2:**
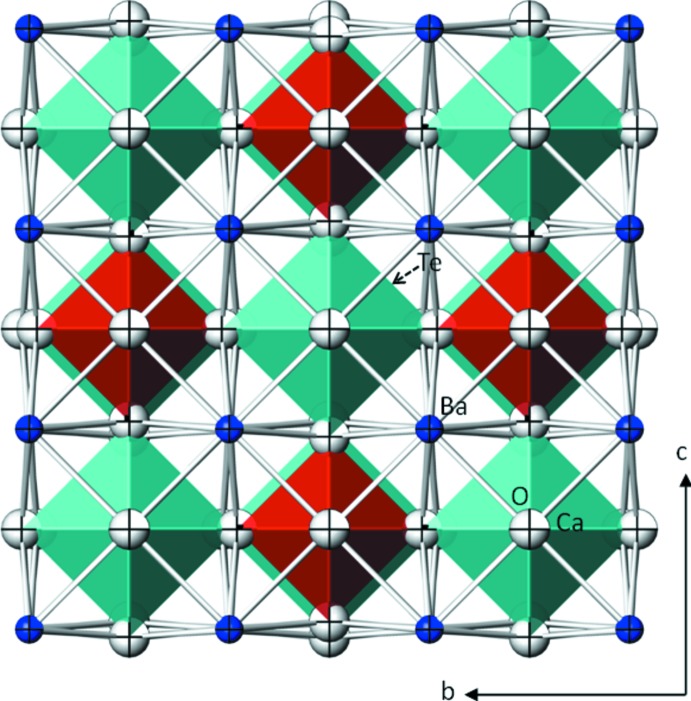
Projection of the crystal structure of Ba_2_CaTeO_6_ (III) along [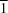
00]. [CaO_6_] octa­hedra are turquoise, [TeO_6_] octa­hedra are red, Ba sites blue and O sites pale grey. Displacement ellipsoids are drawn at the 97% probability level.

The current refinement of Ba_2_CaTeO_6_ (III) is based on single crystal X-ray data and confirms the previous structure determination from X-ray powder diffraction data, but with higher precision (reliability factors for the previous deter­min­ation: *R*_wp_ = 0.159, *R*_p_ = 0.112; Fu *et al.*, 2008 ▸). Ba_2_CaTeO_6_ (III) is a member of the double perovskite family with general formula *A*_2_^[12*co*]^ *B′* ^[6*o*]^ *B′′* ^[6*o*]^O_6_. Dependent on the cations present at the *B*′ and *B′′* sites, double perovskites are functional oxide materials with inter­esting electronic and magnetic properties (Vasala & Karppinen, 2015 ▸). In the crystal structure of (III), Ba, Ca and Te are located on the *A*, *B*′ and *B′′* sites, respectively. The Wyckoff positions and site symmetries of the four sites present in the structure of (III) are: Ba on 8*c* (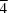
3*m*), Ca on 4*a* (*m* 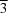 *m*), Te on 4*b* (*m* 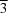 *m*), and O on 24*e* (4*m.m*). Since Ba_2_CaTeO_6_ represents the highest possible symmetry of a double perovskite structure (cubic elpasolite-type in space group type *Fm* 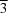 *m*), tilting of the *B*′O_6_ or *B*′′O_6_ octa­hedra (Howard *et al.*, 2003 ▸), like in the monoclinic structure of Sr_2_CaTeO_6_ (Prior *et al.*, 2005 ▸), is not observed. The ordering of the CaO_6_ and TeO_6_ octa­hedra in a checkerboard arrangement in (III) is displayed in Fig. 2 ▸.

With the exception of the Na—O bond length, all other bond lengths (Table 1 ▸) are characteristic for their respective coordination polyhedra and in good agreement with mean values compiled recently for alkali and alkaline earth cations bonded to oxygen: K—O = 2.955 Å for coordination number (CN) 9, 3.095 Å for CN 12; Ca—O = 2.668 Å for CN 12; Ba—O = 2.689 Å for CN 6, 2.965 Å for CN 12 (Gagné & Hawthorne, 2016 ▸). The same is valid for the mean value of octa­hedrally coordinated Te^VI^ with a mean Te—O bond length of 1.923 Å (Gagné & Hawthorne, 2018 ▸). As noted above, the Na—O bond length deviates from the mean value. At 2.3037 (16) Å it is considerably shorter than the mean of 2.441 Å for CN 6 (Gagné & Hawthorne, 2016 ▸). Such a compression has also been reported for other 6*H*-BaTiO_3_-type structures containing sodium. For example, the Na—O distance in K_3_NaOs_2_O_9_ has nearly the same value [2.313 (6) Å; Mogare *et al.*, 2012 ▸] but is reported to be significantly shorter in Ba_3_NaRuIrO_9_ [2.058 (9) Å; Lufaso & zur Loye, 2005*b* ▸].

## Synthesis and crystallization   

Ba[H_4_TeO_6_] was prepared according to a literature protocol (Engelbrecht & Sladky, 1965 ▸) and its purity checked by X-ray powder diffraction. One gram of dried Ba[H_4_TeO_6_] was mixed with five grams of a KNO_3_/KI mixture (stoichiometric ratio 2:1) for (I) or a KNO_3_/NaNO_3_ mixture (stoichiometric ratio 1:1) for (II). The mixtures were placed in platinum crucibles and heated within six h to 773 K, held at that temperature for four days and cooled to room temperature within 12 h. The solidified melts were leached out with water and the remaining solid filtered off, washed with water and ethanol. Colourless single crystals with a hexa­gonal form for both (I) and (II) were selected from the reaction products. In one case a porcelain crucible was used to reproduce the formation of (I). In this batch, very few colourless crystals of Ba_2_CaTeO_6_ (III) had formed as a minor by-product. The porcelain crucible is an adventitious source of calcium that is present in feldspars such as oligoclase used for manufacturing.

## Refinement   

Crystal data, data collection and structure refinement details are summarized in Table 2 ▸. For refinements of (I) and (II) the coordinates of isotypic Ba_3_LaRuO_9_ (Doi *et al.*, 2002 ▸) were used as starting parameters. In the structure of (II), the *M*1 position with site symmetry 3*m*. of Wyckoff site 4*f* is stat­istically occupied by K^+^ and Ba^2+^ cations. For refinement of (III), the starting parameters were taken from the previous sructure determination based on X-ray powder diffraction data (Fu *et al.*, 2008 ▸). The type of element on the metal positions was checked by free refinement of the respective site-occupation factors, which confirmed Ca and Ba, respectively.

## Supplementary Material

Crystal structure: contains datablock(s) I, II, III, global. DOI: 10.1107/S2056989018009064/pj2054sup1.cif

Structure factors: contains datablock(s) I. DOI: 10.1107/S2056989018009064/pj2054Isup2.hkl

Structure factors: contains datablock(s) II. DOI: 10.1107/S2056989018009064/pj2054IIsup3.hkl

Structure factors: contains datablock(s) III. DOI: 10.1107/S2056989018009064/pj2054IIIsup4.hkl

CCDC references: 1850819, 1850818, 1850817

Additional supporting information:  crystallographic information; 3D view; checkCIF report

## Figures and Tables

**Table 1:** Selected bond lengths (Å) and angles (°) in the structures (I)–(III)

(I)		(II)		(III)	
K1—O1	2.893 (2) [3×]	(Ba/K)1—O2	2.98780 (19) [6×]	Ba—O	2.9577 (5) [12×]
K1—O2	3.0359 (17) [6×]	(Ba/K)1—O2	3.1064 (19) [3×]	Ca—O	2.247 (3) [6x]
		(Ba/K)1—O1	3.1927 (12) [3×]	Te—O	1.930 (3) [6×]
Ba1—O2	2.952 (2) [6×]	Ba1—O2	2.9532 (18) [6×]		
Ba1—O1	3.0382 (17) [6×]	Ba1—O1	2.9935 (14) [6×]		
Te1—O2	1.8524 (18) [3×]	Te1—O2	1.8481 (16) [3×]		
Te1—O1	2.0474 (16) [3×]	Te1—O1	2.0418 (14) [3×]		
Ba2—O2	2.5910 (18) [6×]	Na2—O2	2.3037 (16) [6×]		
					
O1—Te1—O1	75.43 (7) [3×]	O1—Te1—O1	75.95 (6) [3×]		
Δ*^*a*^*	0.076	Δ*^*a*^*	0.191		
					

Note: (*a*) Δ is the center shift (Å) of the Te atoms in the Te_2_O_9_ dimer. The center shift is defined as the distance between the Te atoms in the 4*f* Wyckoff position (*z* ≃ 1/6) of the actual crystal structure and the ideal high-symmetry 4*f* Te position (*z* = 1/6) (Lufaso & zur Loye, 2005*a* ▸).

**Table 2:** Experimental details

	(I)	(II)	(III)
Crystal data			
Chemical formula	Ba_2_K_2_Te_2_O_9_	Ba_2_KNaTe_2_O_9_	Ba_2_CaTeO_6_
*M*_r_	752.08	735.97	538.36
Crystal system, space group	Hexagonal, *P*6_3_/*m* *m* *c*	Hexagonal, *P*6_3_/*m* *m* *c*	Cubic, *F* *m* 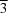 *m*
Temperature (K)	298	293	293
*a*, *b*, *c* (Å)	6.047 (3), 6.047 (3), 16.479 (9)	5.9625 (3), 5.9625 (3), 14.9396 (8)	8.3536 (14), 8.3536 (14), 8.3536 (14)
α, β, γ (°)	90, 90, 120	90, 90, 120	90, 90, 90
*V* (Å^3^)	521.8 (6)	459.97 (5)	582.9 (3)
*Z*	2	2	4
Radiation type	Mo *K*α	Mo *K*α	Mo *K*α
μ (mm^−1^)	13.80	15.25	19.18
Crystal size (mm)	0.09 × 0.09 × 0.01	0.10 × 0.10 × 0.01	0.08 × 0.08 × 0.08

Data collection			
Diffractometer	Bruker APEXII CCD	Bruker APEXII CCD	Bruker APEXII CCD
Absorption correction	Multi-scan (*SADABS*; Bruker, 2015)	Multi-scan (*SADABS*; Bruker, 2015)	Multi-scan (*SADABS*; Bruker, 2015)
*T*_min_, *T*_max_	0.488, 0.748	0.540, 0.749	0.514, 0.748
No. of measured, independent and observed [*I* > 2σ(*I*)] reflections	21821, 754, 676	11701, 702, 669	11194, 131, 131
*R*_int_	0.041	0.029	0.139
(sin θ/λ)_max_ (Å^−1^)	0.943	0.961	0.919

Refinement			
*R*[*F* ^2^ > 2σ(*F* ^2^)], *wR*(*F* ^2^), *S*	0.015, 0.036, 1.15	0.018, 0.034, 1.49	0.019, 0.049, 1.33
No. of reflections	754	702	131
No. of parameters	22	23	7
Δρ_max_, Δρ_min_ (e Å^−3^)	2.90, −2.02	1.02, −1.63	3.87, −1.68

Computer programs: *APEX2* and *SAINT* (Bruker, 2015 ▸), *SHELXL2017* (Sheldrick, 2015 ▸), *ATOMS for Windows* (Dowty, 2006 ▸) and *publCIF* (Westrip, 2010 ▸).

## supplementary crystallographic information

### Dibarium dipotassium nonaoxidoditellurate (I) Crystal data

**Table:**

Ba_2_K_2_Te_2_O_9_	*D*_x_ = 4.786 Mg m^−^^3^
*M**_r_* = 752.08	Mo *K*α radiation, λ = 0.71073 Å
Hexagonal, *P*6_3_/*m**m**c*	Cell parameters from 9371 reflections
*a* = 6.047 (3) Å	θ = 3.9–42.1°
*c* = 16.479 (9) Å	µ = 13.80 mm^−^^1^
*V* = 521.8 (6) Å^3^	*T* = 298 K
*Z* = 2	Plate, colourless
*F*(000) = 652	0.09 × 0.09 × 0.01 mm

### Dibarium dipotassium nonaoxidoditellurate (I) Data collection

**Table:**

Bruker APEXII CCD diffractometer	676 reflections with *I* > 2σ(*I*)
ω– and φ–scans	*R*_int_ = 0.041
Absorption correction: multi-scan (*SADABS*; Bruker, 2015)	θ_max_ = 42.1°, θ_min_ = 3.9°
*T*_min_ = 0.488, *T*_max_ = 0.748	*h* = −11→11
21821 measured reflections	*k* = −11→11
754 independent reflections	*l* = −31→30

### Dibarium dipotassium nonaoxidoditellurate (I) Refinement

**Table:**

Refinement on *F*^2^	0 restraints
Least-squares matrix: full	*w* = 1/[σ^2^(*F*_o_^2^) + (0.0151*P*)^2^ + 0.8791*P*] where *P* = (*F*_o_^2^ + 2*F*_c_^2^)/3
*R*[*F*^2^ > 2σ(*F*^2^)] = 0.015	(Δ/σ)_max_ < 0.001
*wR*(*F*^2^) = 0.036	Δρ_max_ = 2.90 e Å^−^^3^
*S* = 1.15	Δρ_min_ = −2.02 e Å^−^^3^
754 reflections	Extinction correction: SHELXL2014 (Sheldrick, 2015), Fc^*^=kFc[1+0.001xFc^2^λ^3^/sin(2θ)]^-1/4^
22 parameters	Extinction coefficient: 0.0138 (4)

### Dibarium dipotassium nonaoxidoditellurate (I) Special details

**Table:**

Geometry. All esds (except the esd in the dihedral angle between two l.s. planes) are estimated using the full covariance matrix. The cell esds are taken into account individually in the estimation of esds in distances, angles and torsion angles; correlations between esds in cell parameters are only used when they are defined by crystal symmetry. An approximate (isotropic) treatment of cell esds is used for estimating esds involving l.s. planes.

### Dibarium dipotassium nonaoxidoditellurate (I) Fractional atomic coordinates and isotropic or equivalent isotropic displacement parameters (Å^2^)

**Table:**

	*x*	*y*	*z*	*U*_iso_*/*U*_eq_	
K1	0.3333	0.6667	0.87422 (7)	0.02257 (17)	
Ba1	0.0000	0.0000	0.2500	0.00960 (5)	
Te1	0.3333	0.6667	0.16206 (2)	0.00664 (4)	
Ba2	0.0000	0.0000	0.0000	0.00851 (5)	
O1	0.47142 (19)	0.9428 (4)	0.2500	0.0117 (3)	
O2	0.17636 (16)	0.3527 (3)	0.38974 (10)	0.0198 (3)	

### Dibarium dipotassium nonaoxidoditellurate (I) Atomic displacement parameters (Å^2^)

**Table:**

	*U*^11^	*U*^22^	*U*^33^	*U*^12^	*U*^13^	*U*^23^
K1	0.0158 (2)	0.0158 (2)	0.0360 (5)	0.00792 (10)	0.000	0.000
Ba1	0.00959 (6)	0.00959 (6)	0.00961 (9)	0.00480 (3)	0.000	0.000
Te1	0.00661 (5)	0.00661 (5)	0.00671 (7)	0.00330 (3)	0.000	0.000
Ba2	0.00995 (6)	0.00995 (6)	0.00562 (8)	0.00498 (3)	0.000	0.000
O1	0.0150 (6)	0.0068 (7)	0.0106 (7)	0.0034 (3)	0.000	0.000
O2	0.0264 (6)	0.0116 (6)	0.0165 (6)	0.0058 (3)	0.0037 (3)	0.0075 (5)

### Dibarium dipotassium nonaoxidoditellurate (I) Geometric parameters (Å, º)

**Table:**

K1—O1^i^	2.893 (2)	Ba1—O1^xvii^	3.0382 (17)
K1—O1^ii^	2.893 (2)	Ba1—O1^xviii^	3.0382 (17)
K1—O1^iii^	2.893 (2)	Ba1—O1^xix^	3.0382 (17)
K1—O2^iv^	3.0359 (17)	Ba1—O1^xx^	3.0382 (17)
K1—O2^v^	3.0359 (17)	Te1—O2^xiii^	1.8524 (18)
K1—O2^vi^	3.0359 (17)	Te1—O2^xxi^	1.8524 (18)
K1—O2^vii^	3.0359 (17)	Te1—O2^xxii^	1.8525 (18)
K1—O2^viii^	3.0360 (17)	Te1—O1	2.0474 (16)
K1—O2^ix^	3.0360 (17)	Te1—O1^xvii^	2.0474 (16)
Ba1—O2^x^	2.952 (2)	Te1—O1^xv^	2.0474 (16)
Ba1—O2^xi^	2.952 (2)	Ba2—O2^x^	2.5910 (18)
Ba1—O2	2.952 (2)	Ba2—O2^xxiii^	2.5910 (18)
Ba1—O2^xii^	2.952 (2)	Ba2—O2^xiii^	2.5911 (18)
Ba1—O2^xiii^	2.952 (2)	Ba2—O2^xxiv^	2.5911 (18)
Ba1—O2^xiv^	2.952 (2)	Ba2—O2^xii^	2.5911 (18)
Ba1—O1^xv^	3.0382 (17)	Ba2—O2^xxv^	2.5911 (18)
Ba1—O1^xvi^	3.0382 (17)		
			
O1^i^—K1—O1^ii^	75.48 (6)	O1^xvi^—Ba1—O1^xviii^	48.69 (7)
O1^i^—K1—O1^iii^	75.48 (6)	O1^xvii^—Ba1—O1^xviii^	71.31 (7)
O1^ii^—K1—O1^iii^	75.48 (6)	O2^x^—Ba1—O1^xix^	55.25 (3)
O1^i^—K1—O2^iv^	94.78 (4)	O2^xi^—Ba1—O1^xix^	55.25 (3)
O1^ii^—K1—O2^iv^	55.82 (4)	O2—Ba1—O1^xix^	120.56 (3)
O1^iii^—K1—O2^iv^	131.10 (5)	O2^xii^—Ba1—O1^xix^	93.53 (2)
O1^i^—K1—O2^v^	55.82 (4)	O2^xiii^—Ba1—O1^xix^	120.56 (3)
O1^ii^—K1—O2^v^	94.78 (4)	O2^xiv^—Ba1—O1^xix^	93.53 (2)
O1^iii^—K1—O2^v^	131.10 (5)	O1^xv^—Ba1—O1^xix^	120.0
O2^iv^—K1—O2^v^	63.59 (6)	O1^xvi^—Ba1—O1^xix^	71.31 (8)
O1^i^—K1—O2^vi^	131.10 (5)	O1^xvii^—Ba1—O1^xix^	168.69 (8)
O1^ii^—K1—O2^vi^	55.82 (4)	O1^xviii^—Ba1—O1^xix^	120.0
O1^iii^—K1—O2^vi^	94.77 (4)	O2^x^—Ba1—O1^xx^	55.25 (4)
O2^iv^—K1—O2^vi^	55.94 (7)	O2^xi^—Ba1—O1^xx^	55.25 (3)
O2^v^—K1—O2^vi^	119.299 (12)	O2—Ba1—O1^xx^	93.53 (2)
O1^i^—K1—O2^vii^	55.82 (4)	O2^xii^—Ba1—O1^xx^	120.56 (3)
O1^ii^—K1—O2^vii^	131.10 (5)	O2^xiii^—Ba1—O1^xx^	93.53 (2)
O1^iii^—K1—O2^vii^	94.78 (4)	O2^xiv^—Ba1—O1^xx^	120.56 (3)
O2^iv^—K1—O2^vii^	119.299 (12)	O1^xv^—Ba1—O1^xx^	71.31 (8)
O2^v^—K1—O2^vii^	55.94 (7)	O1^xvi^—Ba1—O1^xx^	120.0
O2^vi^—K1—O2^vii^	169.60 (8)	O1^xvii^—Ba1—O1^xx^	120.0
O1^i^—K1—O2^viii^	131.10 (5)	O1^xviii^—Ba1—O1^xx^	168.69 (8)
O1^ii^—K1—O2^viii^	94.78 (4)	O1^xix^—Ba1—O1^xx^	48.69 (8)
O1^iii^—K1—O2^viii^	55.82 (4)	O2^xiii^—Te1—O2^xxi^	100.46 (7)
O2^iv^—K1—O2^viii^	119.298 (12)	O2^xiii^—Te1—O2^xxii^	100.45 (7)
O2^v^—K1—O2^viii^	169.60 (8)	O2^xxi^—Te1—O2^xxii^	100.45 (7)
O2^vi^—K1—O2^viii^	63.59 (6)	O2^xiii^—Te1—O1	162.38 (7)
O2^vii^—K1—O2^viii^	119.297 (12)	O2^xxi^—Te1—O1	90.73 (6)
O1^i^—K1—O2^ix^	94.78 (4)	O2^xxii^—Te1—O1	90.73 (6)
O1^ii^—K1—O2^ix^	131.10 (5)	O2^xiii^—Te1—O1^xvii^	90.73 (6)
O1^iii^—K1—O2^ix^	55.82 (4)	O2^xxi^—Te1—O1^xvii^	162.38 (7)
O2^iv^—K1—O2^ix^	169.60 (8)	O2^xxii^—Te1—O1^xvii^	90.73 (6)
O2^v^—K1—O2^ix^	119.298 (12)	O1—Te1—O1^xvii^	75.43 (7)
O2^vi^—K1—O2^ix^	119.297 (12)	O2^xiii^—Te1—O1^xv^	90.73 (6)
O2^vii^—K1—O2^ix^	63.59 (6)	O2^xxi^—Te1—O1^xv^	90.73 (6)
O2^viii^—K1—O2^ix^	55.93 (7)	O2^xxii^—Te1—O1^xv^	162.38 (7)
O2^x^—Ba1—O2^xi^	102.53 (7)	O1—Te1—O1^xv^	75.43 (7)
O2^x^—Ba1—O2	143.54 (3)	O1^xvii^—Te1—O1^xv^	75.43 (7)
O2^xi^—Ba1—O2	65.62 (6)	O2^x^—Ba2—O2^xxiii^	180.00 (5)
O2^x^—Ba1—O2^xii^	65.62 (6)	O2^x^—Ba2—O2^xiii^	76.25 (7)
O2^xi^—Ba1—O2^xii^	143.54 (3)	O2^xxiii^—Ba2—O2^xiii^	103.75 (7)
O2—Ba1—O2^xii^	143.54 (3)	O2^x^—Ba2—O2^xxiv^	103.75 (7)
O2^x^—Ba1—O2^xiii^	65.62 (6)	O2^xxiii^—Ba2—O2^xxiv^	76.25 (7)
O2^xi^—Ba1—O2^xiii^	143.54 (3)	O2^xiii^—Ba2—O2^xxiv^	180.00 (8)
O2—Ba1—O2^xiii^	102.53 (7)	O2^x^—Ba2—O2^xii^	76.25 (7)
O2^xii^—Ba1—O2^xiii^	65.62 (6)	O2^xxiii^—Ba2—O2^xii^	103.75 (7)
O2^x^—Ba1—O2^xiv^	143.54 (3)	O2^xiii^—Ba2—O2^xii^	76.25 (7)
O2^xi^—Ba1—O2^xiv^	65.62 (6)	O2^xxiv^—Ba2—O2^xii^	103.75 (7)
O2—Ba1—O2^xiv^	65.62 (6)	O2^x^—Ba2—O2^xxv^	103.75 (7)
O2^xii^—Ba1—O2^xiv^	102.53 (7)	O2^xxiii^—Ba2—O2^xxv^	76.25 (7)
O2^xiii^—Ba1—O2^xiv^	143.54 (3)	O2^xiii^—Ba2—O2^xxv^	103.75 (7)
O2^x^—Ba1—O1^xv^	93.53 (2)	O2^xxiv^—Ba2—O2^xxv^	76.25 (7)
O2^xi^—Ba1—O1^xv^	93.53 (2)	O2^xii^—Ba2—O2^xxv^	180.00 (5)
O2—Ba1—O1^xv^	55.25 (3)	Te1^xiii^—O1—Te1	90.11 (9)
O2^xii^—Ba1—O1^xv^	120.56 (3)	Te1^xiii^—O1—K1^xxvi^	89.91 (5)
O2^xiii^—Ba1—O1^xv^	55.25 (4)	Te1—O1—K1^xxvi^	179.97 (5)
O2^xiv^—Ba1—O1^xv^	120.56 (3)	Te1^xiii^—O1—K1^ii^	179.97 (12)
O2^x^—Ba1—O1^xvi^	93.53 (2)	Te1—O1—K1^ii^	89.91 (5)
O2^xi^—Ba1—O1^xvi^	93.53 (2)	K1^xxvi^—O1—K1^ii^	90.06 (8)
O2—Ba1—O1^xvi^	120.56 (3)	Te1^xiii^—O1—Ba1^xxvii^	93.99 (2)
O2^xii^—Ba1—O1^xvi^	55.25 (3)	Te1—O1—Ba1^xxvii^	93.99 (2)
O2^xiii^—Ba1—O1^xvi^	120.56 (3)	K1^xxvi^—O1—Ba1^xxvii^	86.01 (3)
O2^xiv^—Ba1—O1^xvi^	55.25 (3)	K1^ii^—O1—Ba1^xxvii^	86.01 (3)
O1^xv^—Ba1—O1^xvi^	168.69 (7)	Te1^xiii^—O1—Ba1^xxviii^	93.99 (2)
O2^x^—Ba1—O1^xvii^	120.56 (3)	Te1—O1—Ba1^xxviii^	93.99 (2)
O2^xi^—Ba1—O1^xvii^	120.56 (3)	K1^xxvi^—O1—Ba1^xxviii^	86.01 (3)
O2—Ba1—O1^xvii^	55.25 (3)	K1^ii^—O1—Ba1^xxviii^	86.01 (3)
O2^xii^—Ba1—O1^xvii^	93.53 (2)	Ba1^xxvii^—O1—Ba1^xxviii^	168.69 (7)
O2^xiii^—Ba1—O1^xvii^	55.24 (3)	Te1^xiii^—O2—Ba2^xxix^	162.91 (9)
O2^xiv^—Ba1—O1^xvii^	93.53 (2)	Te1^xiii^—O2—Ba1	101.29 (8)
O1^xv^—Ba1—O1^xvii^	48.69 (8)	Ba2^xxix^—O2—Ba1	95.79 (7)
O1^xvi^—Ba1—O1^xvii^	120.0	Te1^xiii^—O2—K1^xxx^	89.48 (3)
O2^x^—Ba1—O1^xviii^	120.56 (3)	Ba2^xxix^—O2—K1^xxx^	92.02 (3)
O2^xi^—Ba1—O1^xviii^	120.56 (3)	Ba1—O2—K1^xxx^	85.03 (4)
O2—Ba1—O1^xviii^	93.53 (2)	Te1^xiii^—O2—K1^xxxi^	89.48 (3)
O2^xii^—Ba1—O1^xviii^	55.24 (3)	Ba2^xxix^—O2—K1^xxxi^	92.02 (3)
O2^xiii^—Ba1—O1^xviii^	93.53 (2)	Ba1—O2—K1^xxxi^	85.03 (4)
O2^xiv^—Ba1—O1^xviii^	55.24 (3)	K1^xxx^—O2—K1^xxxi^	169.60 (8)
O1^xv^—Ba1—O1^xviii^	120.0		

Symmetry codes: (i) *y*−1, −*x*+*y*, −*z*+1; (ii) −*x*+1, −*y*+2, −*z*+1; (iii) *x*−*y*+1, *x*, −*z*+1; (iv) *y*, −*x*+*y*+1, *z*+1/2; (v) −*x*, −*y*+1, *z*+1/2; (vi) *x*−*y*+1, *x*+1, *z*+1/2; (vii) *x*−*y*, *x*, *z*+1/2; (viii) −*x*+1, −*y*+1, *z*+1/2; (ix) *y*, −*x*+*y*, *z*+1/2; (x) −*x*+*y*, −*x*, −*z*+1/2; (xi) −*x*+*y*, −*x*, *z*; (xii) −*y*, *x*−*y*, −*z*+1/2; (xiii) *x*, *y*, −*z*+1/2; (xiv) −*y*, *x*−*y*, *z*; (xv) −*x*+*y*, −*x*+1, *z*; (xvi) −*x*+*y*−1, −*x*, *z*; (xvii) −*y*+1, *x*−*y*+1, *z*; (xviii) *x*−1, *y*−1, *z*; (xix) −*y*+1, *x*−*y*, *z*; (xx) *x*, *y*−1, *z*; (xxi) −*y*+1, *x*−*y*+1, −*z*+1/2; (xxii) −*x*+*y*, −*x*+1, −*z*+1/2; (xxiii) *x*−*y*, *x*, *z*−1/2; (xxiv) −*x*, −*y*, *z*−1/2; (xxv) *y*, −*x*+*y*, *z*−1/2; (xxvi) −*x*+1, −*y*+2, *z*−1/2; (xxvii) *x*, *y*+1, *z*; (xxviii) *x*+1, *y*+1, *z*; (xxix) −*x*, −*y*, *z*+1/2; (xxx) −*x*+1, −*y*+1, *z*−1/2; (xxxi) −*x*, −*y*+1, *z*−1/2.

### Dibarium potassium sodium nonaoxidoditellurate (II) Crystal data

**Table:**

Ba_2_KNaTe_2_O_9_	*D*_x_ = 5.314 Mg m^−^^3^
*M**_r_* = 735.97	Mo *K*α radiation, λ = 0.71073 Å
Hexagonal, *P*6_3_/*m**m**c*	Cell parameters from 9985 reflections
*a* = 5.9625 (3) Å	θ = 2.7–42.9°
*c* = 14.9396 (8) Å	µ = 15.25 mm^−^^1^
*V* = 459.97 (5) Å^3^	*T* = 293 K
*Z* = 2	Plate, colourless
*F*(000) = 636	0.10 × 0.10 × 0.01 mm

### Dibarium potassium sodium nonaoxidoditellurate (II) Data collection

**Table:**

Bruker APEXII CCD diffractometer	669 reflections with *I* > 2σ(*I*)
ω– and φ–scans	*R*_int_ = 0.029
Absorption correction: multi-scan (*SADABS*; Bruker, 2015)	θ_max_ = 43.1°, θ_min_ = 2.7°
*T*_min_ = 0.540, *T*_max_ = 0.749	*h* = −11→11
11701 measured reflections	*k* = −11→8
702 independent reflections	*l* = −28→28

### Dibarium potassium sodium nonaoxidoditellurate (II) Refinement

**Table:**

Refinement on *F*^2^	0 restraints
Least-squares matrix: full	*w* = 1/[σ^2^(*F*_o_^2^) + (0.0075*P*)^2^ + 0.5711*P*] where *P* = (*F*_o_^2^ + 2*F*_c_^2^)/3
*R*[*F*^2^ > 2σ(*F*^2^)] = 0.018	(Δ/σ)_max_ = 0.001
*wR*(*F*^2^) = 0.034	Δρ_max_ = 1.02 e Å^−^^3^
*S* = 1.49	Δρ_min_ = −1.63 e Å^−^^3^
702 reflections	Extinction correction: SHELXL2014 (Sheldrick, 2015), Fc^*^=kFc[1+0.001xFc^2^λ^3^/sin(2θ)]^-1/4^
23 parameters	Extinction coefficient: 0.0409 (10)

### Dibarium potassium sodium nonaoxidoditellurate (II) Special details

**Table:**

Geometry. All esds (except the esd in the dihedral angle between two l.s. planes) are estimated using the full covariance matrix. The cell esds are taken into account individually in the estimation of esds in distances, angles and torsion angles; correlations between esds in cell parameters are only used when they are defined by crystal symmetry. An approximate (isotropic) treatment of cell esds is used for estimating esds involving l.s. planes.

### Dibarium potassium sodium nonaoxidoditellurate (II) Fractional atomic coordinates and isotropic or equivalent isotropic displacement parameters (Å^2^)

**Table:**

	*x*	*y*	*z*	*U*_iso_*/*U*_eq_	Occ. (<1)
BaK1	0.3333	0.6667	0.91703 (2)	0.01251 (6)	0.5
KBA1	0.3333	0.6667	0.91703 (2)	0.01251 (6)	0.5
Ba1	0.0000	0.0000	0.2500	0.00953 (5)	
Te1	0.3333	0.6667	0.15383 (2)	0.00583 (5)	
Na2	0.0000	0.0000	0.0000	0.0110 (3)	
O1	0.47381 (18)	0.9476 (4)	0.2500	0.0101 (3)	
O2	0.17638 (16)	0.3528 (3)	0.40559 (12)	0.0195 (3)	

### Dibarium potassium sodium nonaoxidoditellurate (II) Atomic displacement parameters (Å^2^)

**Table:**

	*U*^11^	*U*^22^	*U*^33^	*U*^12^	*U*^13^	*U*^23^
BaK1	0.01060 (8)	0.01060 (8)	0.01631 (11)	0.00530 (4)	0.000	0.000
KBA1	0.01060 (8)	0.01060 (8)	0.01631 (11)	0.00530 (4)	0.000	0.000
Ba1	0.00874 (7)	0.00874 (7)	0.01112 (9)	0.00437 (3)	0.000	0.000
Te1	0.00573 (5)	0.00573 (5)	0.00603 (7)	0.00287 (3)	0.000	0.000
Na2	0.0118 (5)	0.0118 (5)	0.0094 (7)	0.0059 (2)	0.000	0.000
O1	0.0107 (5)	0.0060 (6)	0.0121 (6)	0.0030 (3)	0.000	0.000
O2	0.0235 (6)	0.0120 (6)	0.0192 (6)	0.0060 (3)	0.0045 (3)	0.0089 (5)

### Dibarium potassium sodium nonaoxidoditellurate (II) Geometric parameters (Å, º)

**Table:**

BaK1—O2^i^	2.9878 (2)	Ba1—O1^xviii^	2.9935 (14)
BaK1—O2^ii^	2.9878 (2)	Ba1—O1^xix^	2.9935 (14)
BaK1—O2^iii^	2.9878 (2)	Ba1—O1^xx^	2.9935 (14)
BaK1—O2^iv^	2.9878 (2)	Ba1—O1^xxi^	2.9935 (14)
BaK1—O2^v^	2.9878 (2)	Ba1—O1^xxii^	2.9935 (14)
BaK1—O2^vi^	2.9879 (2)	Ba1—O1^xxiii^	2.9935 (14)
BaK1—O2^vii^	3.1064 (18)	Te1—O2^xxiv^	1.8481 (16)
BaK1—O2^viii^	3.1064 (19)	Te1—O2^xiv^	1.8481 (16)
BaK1—O2^ix^	3.1064 (19)	Te1—O2^xxv^	1.8481 (16)
BaK1—O1^x^	3.1927 (12)	Te1—O1^xx^	2.0418 (14)
BaK1—O1^xi^	3.1927 (12)	Te1—O1^xviii^	2.0418 (14)
BaK1—O1^xii^	3.1927 (12)	Te1—O1	2.0418 (14)
Ba1—O2^xiii^	2.9532 (18)	Na2—O2^xiv^	2.3037 (16)
Ba1—O2^xiv^	2.9532 (18)	Na2—O2^xxvi^	2.3037 (16)
Ba1—O2^xv^	2.9532 (18)	Na2—O2^xiii^	2.3037 (16)
Ba1—O2	2.9532 (18)	Na2—O2^xxvii^	2.3037 (16)
Ba1—O2^xvi^	2.9532 (18)	Na2—O2^xvi^	2.3038 (16)
Ba1—O2^xvii^	2.9532 (18)	Na2—O2^xxviii^	2.3038 (16)
			
O2^i^—BaK1—O2^ii^	56.05 (6)	O2^xv^—Ba1—O1^xxii^	93.19 (2)
O2^i^—BaK1—O2^iii^	63.74 (7)	O2—Ba1—O1^xxii^	120.27 (3)
O2^ii^—BaK1—O2^iii^	119.677 (7)	O2^xvi^—Ba1—O1^xxii^	55.95 (3)
O2^i^—BaK1—O2^iv^	119.677 (7)	O2^xvii^—Ba1—O1^xxii^	55.95 (3)
O2^ii^—BaK1—O2^iv^	63.74 (7)	O1^xviii^—Ba1—O1^xxii^	120.0
O2^iii^—BaK1—O2^iv^	172.40 (6)	O1^xix^—Ba1—O1^xxii^	70.37 (7)
O2^i^—BaK1—O2^v^	119.676 (7)	O1^xx^—Ba1—O1^xxii^	169.63 (7)
O2^ii^—BaK1—O2^v^	172.40 (6)	O1^xxi^—Ba1—O1^xxii^	120.0
O2^iii^—BaK1—O2^v^	56.04 (6)	O2^xiii^—Ba1—O1^xxiii^	120.27 (3)
O2^iv^—BaK1—O2^v^	119.675 (7)	O2^xiv^—Ba1—O1^xxiii^	93.19 (2)
O2^i^—BaK1—O2^vi^	172.40 (6)	O2^xv^—Ba1—O1^xxiii^	120.27 (3)
O2^ii^—BaK1—O2^vi^	119.676 (7)	O2—Ba1—O1^xxiii^	93.20 (2)
O2^iii^—BaK1—O2^vi^	119.675 (7)	O2^xvi^—Ba1—O1^xxiii^	55.95 (3)
O2^iv^—BaK1—O2^vi^	56.04 (6)	O2^xvii^—Ba1—O1^xxiii^	55.95 (3)
O2^v^—BaK1—O2^vi^	63.74 (6)	O1^xviii^—Ba1—O1^xxiii^	70.37 (7)
O2^i^—BaK1—O2^vii^	120.56 (3)	O1^xix^—Ba1—O1^xxiii^	120.0
O2^ii^—BaK1—O2^vii^	120.56 (3)	O1^xx^—Ba1—O1^xxiii^	120.0
O2^iii^—BaK1—O2^vii^	91.79 (4)	O1^xxi^—Ba1—O1^xxiii^	169.63 (7)
O2^iv^—BaK1—O2^vii^	91.79 (4)	O1^xxii^—Ba1—O1^xxiii^	49.63 (7)
O2^v^—BaK1—O2^vii^	66.84 (5)	O2^xxiv^—Te1—O2^xiv^	98.85 (7)
O2^vi^—BaK1—O2^vii^	66.84 (5)	O2^xxiv^—Te1—O2^xxv^	98.85 (7)
O2^i^—BaK1—O2^viii^	91.79 (4)	O2^xiv^—Te1—O2^xxv^	98.85 (7)
O2^ii^—BaK1—O2^viii^	66.84 (5)	O2^xxiv^—Te1—O1^xx^	91.52 (5)
O2^iii^—BaK1—O2^viii^	120.56 (3)	O2^xiv^—Te1—O1^xx^	91.51 (5)
O2^iv^—BaK1—O2^viii^	66.84 (5)	O2^xxv^—Te1—O1^xx^	163.99 (7)
O2^v^—BaK1—O2^viii^	120.56 (3)	O2^xxiv^—Te1—O1^xviii^	163.99 (7)
O2^vi^—BaK1—O2^viii^	91.79 (4)	O2^xiv^—Te1—O1^xviii^	91.51 (5)
O2^vii^—BaK1—O2^viii^	53.73 (5)	O2^xxv^—Te1—O1^xviii^	91.51 (5)
O2^i^—BaK1—O2^ix^	66.84 (5)	O1^xx^—Te1—O1^xviii^	75.95 (6)
O2^ii^—BaK1—O2^ix^	91.79 (4)	O2^xxiv^—Te1—O1	91.51 (5)
O2^iii^—BaK1—O2^ix^	66.84 (5)	O2^xiv^—Te1—O1	163.99 (7)
O2^iv^—BaK1—O2^ix^	120.56 (3)	O2^xxv^—Te1—O1	91.52 (5)
O2^v^—BaK1—O2^ix^	91.79 (4)	O1^xx^—Te1—O1	75.95 (6)
O2^vi^—BaK1—O2^ix^	120.56 (3)	O1^xviii^—Te1—O1	75.95 (6)
O2^vii^—BaK1—O2^ix^	53.73 (5)	O2^xiv^—Na2—O2^xxvi^	180.00 (9)
O2^viii^—BaK1—O2^ix^	53.73 (5)	O2^xiv^—Na2—O2^xiii^	86.43 (7)
O2^i^—BaK1—O1^x^	88.64 (4)	O2^xxvi^—Na2—O2^xiii^	93.57 (7)
O2^ii^—BaK1—O1^x^	118.94 (4)	O2^xiv^—Na2—O2^xxvii^	93.57 (7)
O2^iii^—BaK1—O1^x^	53.54 (4)	O2^xxvi^—Na2—O2^xxvii^	86.43 (7)
O2^iv^—BaK1—O1^x^	118.94 (4)	O2^xiii^—Na2—O2^xxvii^	180.00 (9)
O2^v^—BaK1—O1^x^	53.54 (4)	O2^xiv^—Na2—O2^xvi^	86.43 (7)
O2^vi^—BaK1—O1^x^	88.64 (4)	O2^xxvi^—Na2—O2^xvi^	93.57 (7)
O2^vii^—BaK1—O1^x^	120.26 (3)	O2^xiii^—Na2—O2^xvi^	86.43 (7)
O2^viii^—BaK1—O1^x^	172.86 (4)	O2^xxvii^—Na2—O2^xvi^	93.57 (7)
O2^ix^—BaK1—O1^x^	120.26 (3)	O2^xiv^—Na2—O2^xxviii^	93.57 (7)
O2^i^—BaK1—O1^xi^	118.94 (4)	O2^xxvi^—Na2—O2^xxviii^	86.43 (7)
O2^ii^—BaK1—O1^xi^	88.64 (4)	O2^xiii^—Na2—O2^xxviii^	93.57 (7)
O2^iii^—BaK1—O1^xi^	118.94 (4)	O2^xxvii^—Na2—O2^xxviii^	86.43 (7)
O2^iv^—BaK1—O1^xi^	53.54 (4)	O2^xvi^—Na2—O2^xxviii^	180.00 (7)
O2^v^—BaK1—O1^xi^	88.64 (4)	Te1^xiv^—O1—Te1	89.44 (8)
O2^vi^—BaK1—O1^xi^	53.54 (4)	Te1^xiv^—O1—Ba1^xxix^	93.68 (2)
O2^vii^—BaK1—O1^xi^	120.26 (3)	Te1—O1—Ba1^xxix^	93.68 (2)
O2^viii^—BaK1—O1^xi^	120.26 (3)	Te1^xiv^—O1—Ba1^xxx^	93.68 (2)
O2^ix^—BaK1—O1^xi^	172.86 (4)	Te1—O1—Ba1^xxx^	93.68 (2)
O1^x^—BaK1—O1^xi^	65.40 (4)	Ba1^xxix^—O1—Ba1^xxx^	169.63 (7)
O2^i^—BaK1—O1^xii^	53.54 (4)	Te1^xiv^—O1—BaK1^xxxi^	83.874 (13)
O2^ii^—BaK1—O1^xii^	53.54 (4)	Te1—O1—BaK1^xxxi^	173.32 (6)
O2^iii^—BaK1—O1^xii^	88.64 (4)	Ba1^xxix^—O1—BaK1^xxxi^	86.77 (2)
O2^iv^—BaK1—O1^xii^	88.64 (4)	Ba1^xxx^—O1—BaK1^xxxi^	86.77 (2)
O2^v^—BaK1—O1^xii^	118.94 (4)	Te1^xiv^—O1—KBA1^xxxi^	83.874 (13)
O2^vi^—BaK1—O1^xii^	118.94 (4)	Te1—O1—KBA1^xxxi^	173.32 (6)
O2^vii^—BaK1—O1^xii^	172.86 (4)	Ba1^xxix^—O1—KBA1^xxxi^	86.77 (2)
O2^viii^—BaK1—O1^xii^	120.26 (3)	Ba1^xxx^—O1—KBA1^xxxi^	86.77 (2)
O2^ix^—BaK1—O1^xii^	120.26 (3)	BaK1^xxxi^—O1—KBA1^xxxi^	0.000 (7)
O1^x^—BaK1—O1^xii^	65.40 (4)	Te1^xiv^—O1—BaK1^xi^	173.32 (6)
O1^xi^—BaK1—O1^xii^	65.40 (4)	Te1—O1—BaK1^xi^	83.874 (13)
O2^xiii^—Ba1—O2^xiv^	64.58 (5)	Ba1^xxix^—O1—BaK1^xi^	86.77 (2)
O2^xiii^—Ba1—O2^xv^	103.83 (6)	Ba1^xxx^—O1—BaK1^xi^	86.77 (2)
O2^xiv^—Ba1—O2^xv^	144.07 (2)	BaK1^xxxi^—O1—BaK1^xi^	102.81 (5)
O2^xiii^—Ba1—O2	144.07 (3)	KBA1^xxxi^—O1—BaK1^xi^	102.8
O2^xiv^—Ba1—O2	103.83 (6)	Te1^xiv^—O1—KBA1^xi^	173.32 (6)
O2^xv^—Ba1—O2	64.58 (5)	Te1—O1—KBA1^xi^	83.874 (13)
O2^xiii^—Ba1—O2^xvi^	64.58 (5)	Ba1^xxix^—O1—KBA1^xi^	86.77 (2)
O2^xiv^—Ba1—O2^xvi^	64.58 (5)	Ba1^xxx^—O1—KBA1^xi^	86.77 (2)
O2^xv^—Ba1—O2^xvi^	144.07 (2)	BaK1^xxxi^—O1—KBA1^xi^	102.81 (5)
O2—Ba1—O2^xvi^	144.07 (2)	KBA1^xxxi^—O1—KBA1^xi^	102.81 (5)
O2^xiii^—Ba1—O2^xvii^	144.07 (3)	BaK1^xi^—O1—KBA1^xi^	0.000 (7)
O2^xiv^—Ba1—O2^xvii^	144.07 (3)	Te1^xiv^—O2—Na2^xxxii^	170.96 (10)
O2^xv^—Ba1—O2^xvii^	64.58 (5)	Te1^xiv^—O2—Ba1	99.37 (7)
O2—Ba1—O2^xvii^	64.58 (5)	Na2^xxxii^—O2—Ba1	89.67 (6)
O2^xvi^—Ba1—O2^xvii^	103.83 (6)	Te1^xiv^—O2—BaK1^xxxiii^	93.26 (3)
O2^xiii^—Ba1—O1^xviii^	120.27 (3)	Na2^xxxii^—O2—BaK1^xxxiii^	86.47 (3)
O2^xiv^—Ba1—O1^xviii^	55.95 (3)	Ba1—O2—BaK1^xxxiii^	91.39 (4)
O2^xv^—Ba1—O1^xviii^	120.27 (3)	Te1^xiv^—O2—KBA1^xxxiii^	93.26 (3)
O2—Ba1—O1^xviii^	55.95 (3)	Na2^xxxii^—O2—KBA1^xxxiii^	86.47 (3)
O2^xvi^—Ba1—O1^xviii^	93.20 (2)	Ba1—O2—KBA1^xxxiii^	91.39 (4)
O2^xvii^—Ba1—O1^xviii^	93.19 (2)	BaK1^xxxiii^—O2—KBA1^xxxiii^	0.000 (13)
O2^xiii^—Ba1—O1^xix^	55.95 (3)	Te1^xiv^—O2—BaK1^xxxiv^	93.26 (3)
O2^xiv^—Ba1—O1^xix^	120.27 (3)	Na2^xxxii^—O2—BaK1^xxxiv^	86.47 (3)
O2^xv^—Ba1—O1^xix^	55.95 (3)	Ba1—O2—BaK1^xxxiv^	91.39 (4)
O2—Ba1—O1^xix^	120.27 (3)	BaK1^xxxiii^—O2—BaK1^xxxiv^	172.40 (6)
O2^xvi^—Ba1—O1^xix^	93.20 (2)	KBA1^xxxiii^—O2—BaK1^xxxiv^	172.4
O2^xvii^—Ba1—O1^xix^	93.20 (2)	Te1^xiv^—O2—KBA1^xxxiv^	93.26 (3)
O1^xviii^—Ba1—O1^xix^	169.63 (7)	Na2^xxxii^—O2—KBA1^xxxiv^	86.47 (3)
O2^xiii^—Ba1—O1^xx^	93.20 (2)	Ba1—O2—KBA1^xxxiv^	91.39 (4)
O2^xiv^—Ba1—O1^xx^	55.95 (3)	BaK1^xxxiii^—O2—KBA1^xxxiv^	172.40 (6)
O2^xv^—Ba1—O1^xx^	93.20 (2)	KBA1^xxxiii^—O2—KBA1^xxxiv^	172.40 (6)
O2—Ba1—O1^xx^	55.95 (3)	BaK1^xxxiv^—O2—KBA1^xxxiv^	0.0
O2^xvi^—Ba1—O1^xx^	120.27 (3)	Te1^xiv^—O2—KBA1^ix^	87.26 (6)
O2^xvii^—Ba1—O1^xx^	120.27 (3)	Na2^xxxii^—O2—KBA1^ix^	83.70 (5)
O1^xviii^—Ba1—O1^xx^	49.63 (7)	Ba1—O2—KBA1^ix^	173.37 (6)
O1^xix^—Ba1—O1^xx^	120.0	BaK1^xxxiii^—O2—KBA1^ix^	88.21 (4)
O2^xiii^—Ba1—O1^xxi^	55.95 (3)	KBA1^xxxiii^—O2—KBA1^ix^	88.21 (4)
O2^xiv^—Ba1—O1^xxi^	93.20 (2)	BaK1^xxxiv^—O2—KBA1^ix^	88.21 (4)
O2^xv^—Ba1—O1^xxi^	55.95 (3)	KBA1^xxxiv^—O2—KBA1^ix^	88.21 (4)
O2—Ba1—O1^xxi^	93.20 (2)	Te1^xiv^—O2—BaK1^ix^	87.26 (6)
O2^xvi^—Ba1—O1^xxi^	120.27 (3)	Na2^xxxii^—O2—BaK1^ix^	83.70 (5)
O2^xvii^—Ba1—O1^xxi^	120.27 (3)	Ba1—O2—BaK1^ix^	173.37 (6)
O1^xviii^—Ba1—O1^xxi^	120.0	BaK1^xxxiii^—O2—BaK1^ix^	88.21 (4)
O1^xix^—Ba1—O1^xxi^	49.63 (7)	KBA1^xxxiii^—O2—BaK1^ix^	88.2
O1^xx^—Ba1—O1^xxi^	70.37 (7)	BaK1^xxxiv^—O2—BaK1^ix^	88.21 (4)
O2^xiii^—Ba1—O1^xxii^	93.19 (2)	KBA1^xxxiv^—O2—BaK1^ix^	88.2
O2^xiv^—Ba1—O1^xxii^	120.27 (3)	KBA1^ix^—O2—BaK1^ix^	0.0

Symmetry codes: (i) *y*, −*x*+*y*, *z*+1/2; (ii) −*x*+1, −*y*+1, *z*+1/2; (iii) *x*−*y*, *x*, *z*+1/2; (iv) *x*−*y*+1, *x*+1, *z*+1/2; (v) −*x*, −*y*+1, *z*+1/2; (vi) *y*, −*x*+*y*+1, *z*+1/2; (vii) −*x*+*y*, −*x*+1, −*z*+3/2; (viii) −*y*+1, *x*−*y*+1, −*z*+3/2; (ix) *x*, *y*, −*z*+3/2; (x) *y*−1, −*x*+*y*, −*z*+1; (xi) −*x*+1, −*y*+2, −*z*+1; (xii) *x*−*y*+1, *x*, −*z*+1; (xiii) −*y*, *x*−*y*, −*z*+1/2; (xiv) *x*, *y*, −*z*+1/2; (xv) −*y*, *x*−*y*, *z*; (xvi) −*x*+*y*, −*x*, −*z*+1/2; (xvii) −*x*+*y*, −*x*, *z*; (xviii) −*x*+*y*, −*x*+1, *z*; (xix) −*x*+*y*−1, −*x*, *z*; (xx) −*y*+1, *x*−*y*+1, *z*; (xxi) *x*−1, *y*−1, *z*; (xxii) −*y*+1, *x*−*y*, *z*; (xxiii) *x*, *y*−1, *z*; (xxiv) −*x*+*y*, −*x*+1, −*z*+1/2; (xxv) −*y*+1, *x*−*y*+1, −*z*+1/2; (xxvi) −*x*, −*y*, *z*−1/2; (xxvii) *y*, −*x*+*y*, *z*−1/2; (xxviii) *x*−*y*, *x*, *z*−1/2; (xxix) *x*+1, *y*+1, *z*; (xxx) *x*, *y*+1, *z*; (xxxi) −*x*+1, −*y*+2, *z*−1/2; (xxxii) −*x*, −*y*, *z*+1/2; (xxxiii) −*x*+1, −*y*+1, *z*−1/2; (xxxiv) −*x*, −*y*+1, *z*−1/2.

### Dibarium calcium hexaoxidotellurate (III) Crystal data

**Table:**

Ba_2_CaTeO_6_	Mo *K*α radiation, λ = 0.71073 Å
*M**_r_* = 538.36	Cell parameters from 5256 reflections
Cubic, *F**m*3*m*	θ = 4.2–40.4°
*a* = 8.3536 (14) Å	µ = 19.18 mm^−^^1^
*V* = 582.9 (3) Å^3^	*T* = 293 K
*Z* = 4	Octahedron, colourless
*F*(000) = 928	0.08 × 0.08 × 0.08 mm
*D*_x_ = 6.134 Mg m^−^^3^	

### Dibarium calcium hexaoxidotellurate (III) Data collection

**Table:**

Bruker APEXII CCD diffractometer	131 reflections with *I* > 2σ(*I*)
φ– and ω–scans	*R*_int_ = 0.139
Absorption correction: multi-scan (*SADABS*; Bruker, 2015)	θ_max_ = 40.8°, θ_min_ = 4.2°
*T*_min_ = 0.514, *T*_max_ = 0.748	*h* = −15→15
11194 measured reflections	*k* = −15→15
131 independent reflections	*l* = −15→15

### Dibarium calcium hexaoxidotellurate (III) Refinement

**Table:**

Refinement on *F*^2^	7 parameters
Least-squares matrix: full	0 restraints
*R*[*F*^2^ > 2σ(*F*^2^)] = 0.019	*w* = 1/[σ^2^(*F*_o_^2^) + (0.0237*P*)^2^ + 1.8403*P*] where *P* = (*F*_o_^2^ + 2*F*_c_^2^)/3
*wR*(*F*^2^) = 0.049	(Δ/σ)_max_ < 0.001
*S* = 1.33	Δρ_max_ = 3.87 e Å^−^^3^
131 reflections	Δρ_min_ = −1.68 e Å^−^^3^

### Dibarium calcium hexaoxidotellurate (III) Special details

**Table:**

Geometry. All esds (except the esd in the dihedral angle between two l.s. planes) are estimated using the full covariance matrix. The cell esds are taken into account individually in the estimation of esds in distances, angles and torsion angles; correlations between esds in cell parameters are only used when they are defined by crystal symmetry. An approximate (isotropic) treatment of cell esds is used for estimating esds involving l.s. planes.

### Dibarium calcium hexaoxidotellurate (III) Fractional atomic coordinates and isotropic or equivalent isotropic displacement parameters (Å^2^)

**Table:**

	*x*	*y*	*z*	*U*_iso_*/*U*_eq_	
Ba	0.250000	0.250000	0.250000	0.00957 (13)	
Ca	0.000000	0.000000	0.000000	0.0066 (2)	
Te	0.500000	0.500000	0.500000	0.00602 (13)	
O	0.2690 (4)	0.000000	0.000000	0.0203 (6)	

### Dibarium calcium hexaoxidotellurate (III) Atomic displacement parameters (Å^2^)

**Table:**

	*U*^11^	*U*^22^	*U*^33^	*U*^12^	*U*^13^	*U*^23^
Ba	0.00957 (13)	0.00957 (13)	0.00957 (13)	0.000	0.000	0.000
Ca	0.0066 (2)	0.0066 (2)	0.0066 (2)	0.000	0.000	0.000
Te	0.00602 (13)	0.00602 (13)	0.00602 (13)	0.000	0.000	0.000
O	0.0201 (12)	0.0203 (8)	0.0203 (8)	0.000	0.000	0.000

### Dibarium calcium hexaoxidotellurate (III) Geometric parameters (Å, º)

**Table:**

Ba—O^i^	2.9577 (5)	Ca—O	2.247 (3)
Ba—O^ii^	2.9577 (5)	Ca—O^xii^	2.247 (3)
Ba—O	2.9577 (5)	Ca—O^iv^	2.247 (3)
Ba—O^iii^	2.9577 (5)	Ca—O^xiii^	2.247 (3)
Ba—O^iv^	2.9577 (5)	Ca—O^v^	2.247 (3)
Ba—O^v^	2.9577 (5)	Ca—O^xiv^	2.247 (3)
Ba—O^vi^	2.9577 (5)	Te—O^xv^	1.930 (3)
Ba—O^vii^	2.9577 (5)	Te—O^iii^	1.930 (3)
Ba—O^viii^	2.9577 (5)	Te—O^xvi^	1.930 (3)
Ba—O^ix^	2.9577 (5)	Te—O^i^	1.930 (3)
Ba—O^x^	2.9577 (5)	Te—O^xvii^	1.930 (3)
Ba—O^xi^	2.9577 (5)	Te—O^ii^	1.930 (3)
			
O^i^—Ba—O^ii^	54.95 (11)	O^ii^—Ba—O^xi^	90.165 (7)
O^i^—Ba—O	119.905 (4)	O—Ba—O^xi^	90.165 (7)
O^ii^—Ba—O	173.85 (13)	O^iii^—Ba—O^xi^	119.905 (4)
O^i^—Ba—O^iii^	54.95 (11)	O^iv^—Ba—O^xi^	54.95 (11)
O^ii^—Ba—O^iii^	54.95 (11)	O^v^—Ba—O^xi^	119.905 (4)
O—Ba—O^iii^	119.905 (4)	O^vi^—Ba—O^xi^	64.99 (11)
O^i^—Ba—O^iv^	119.905 (4)	O^vii^—Ba—O^xi^	119.905 (4)
O^ii^—Ba—O^iv^	119.905 (4)	O^viii^—Ba—O^xi^	119.905 (4)
O—Ba—O^iv^	64.99 (11)	O^ix^—Ba—O^xi^	54.95 (11)
O^iii^—Ba—O^iv^	173.85 (13)	O^x^—Ba—O^xi^	173.85 (13)
O^i^—Ba—O^v^	173.85 (13)	O—Ca—O^xii^	90.0
O^ii^—Ba—O^v^	119.905 (4)	O—Ca—O^iv^	90.0
O—Ba—O^v^	64.99 (11)	O^xii^—Ca—O^iv^	180.0
O^iii^—Ba—O^v^	119.905 (4)	O—Ca—O^xiii^	90.0
O^iv^—Ba—O^v^	64.99 (11)	O^xii^—Ca—O^xiii^	90.0
O^i^—Ba—O^vi^	64.99 (11)	O^iv^—Ca—O^xiii^	90.0
O^ii^—Ba—O^vi^	119.905 (4)	O—Ca—O^v^	90.0
O—Ba—O^vi^	54.95 (11)	O^xii^—Ca—O^v^	90.0
O^iii^—Ba—O^vi^	90.165 (7)	O^iv^—Ca—O^v^	90.0
O^iv^—Ba—O^vi^	90.165 (7)	O^xiii^—Ca—O^v^	180.0
O^v^—Ba—O^vi^	119.905 (4)	O—Ca—O^xiv^	180.0
O^i^—Ba—O^vii^	119.905 (4)	O^xii^—Ca—O^xiv^	90.0
O^ii^—Ba—O^vii^	64.99 (11)	O^iv^—Ca—O^xiv^	90.0
O—Ba—O^vii^	119.905 (4)	O^xiii^—Ca—O^xiv^	90.0
O^iii^—Ba—O^vii^	90.165 (7)	O^v^—Ca—O^xiv^	90.0
O^iv^—Ba—O^vii^	90.165 (7)	O^xv^—Te—O^iii^	180.0
O^v^—Ba—O^vii^	54.95 (11)	O^xv^—Te—O^xvi^	90.0
O^vi^—Ba—O^vii^	173.85 (13)	O^iii^—Te—O^xvi^	90.0
O^i^—Ba—O^viii^	90.165 (7)	O^xv^—Te—O^i^	90.0
O^ii^—Ba—O^viii^	119.905 (4)	O^iii^—Te—O^i^	90.0
O—Ba—O^viii^	54.95 (11)	O^xvi^—Te—O^i^	180.0
O^iii^—Ba—O^viii^	64.99 (11)	O^xv^—Te—O^xvii^	90.000 (1)
O^iv^—Ba—O^viii^	119.905 (4)	O^iii^—Te—O^xvii^	90.0
O^v^—Ba—O^viii^	90.165 (7)	O^xvi^—Te—O^xvii^	90.000 (1)
O^vi^—Ba—O^viii^	54.95 (11)	O^i^—Te—O^xvii^	90.0
O^vii^—Ba—O^viii^	119.905 (4)	O^xv^—Te—O^ii^	90.0
O^i^—Ba—O^ix^	90.165 (7)	O^iii^—Te—O^ii^	90.000 (1)
O^ii^—Ba—O^ix^	64.99 (11)	O^xvi^—Te—O^ii^	90.0
O—Ba—O^ix^	119.905 (4)	O^i^—Te—O^ii^	90.000 (1)
O^iii^—Ba—O^ix^	119.905 (4)	O^xvii^—Te—O^ii^	180.0
O^iv^—Ba—O^ix^	54.95 (11)	Te^xviii^—O—Ca	180.0
O^v^—Ba—O^ix^	90.165 (7)	Te^xviii^—O—Ba^xviii^	93.08 (7)
O^vi^—Ba—O^ix^	119.905 (4)	Ca—O—Ba^xviii^	86.92 (7)
O^vii^—Ba—O^ix^	64.99 (11)	Te^xviii^—O—Ba	93.08 (7)
O^viii^—Ba—O^ix^	173.85 (13)	Ca—O—Ba	86.92 (7)
O^i^—Ba—O^x^	119.905 (4)	Ba^xviii^—O—Ba	173.85 (13)
O^ii^—Ba—O^x^	90.165 (7)	Te^xviii^—O—Ba^x^	93.08 (7)
O—Ba—O^x^	90.165 (7)	Ca—O—Ba^x^	86.92 (7)
O^iii^—Ba—O^x^	64.99 (11)	Ba^xviii^—O—Ba^x^	89.835 (7)
O^iv^—Ba—O^x^	119.905 (4)	Ba—O—Ba^x^	89.835 (7)
O^v^—Ba—O^x^	54.95 (11)	Te^xviii^—O—Ba^xi^	93.08 (7)
O^vi^—Ba—O^x^	119.905 (4)	Ca—O—Ba^xi^	86.92 (7)
O^vii^—Ba—O^x^	54.95 (11)	Ba^xviii^—O—Ba^xi^	89.835 (7)
O^viii^—Ba—O^x^	64.99 (11)	Ba—O—Ba^xi^	89.835 (7)
O^ix^—Ba—O^x^	119.905 (4)	Ba^x^—O—Ba^xi^	173.85 (13)
O^i^—Ba—O^xi^	64.99 (11)		

Symmetry codes: (i) *z*+1/2, *x*, *y*+1/2; (ii) *x*, *y*+1/2, *z*+1/2; (iii) *y*+1/2, *z*+1/2, *x*; (iv) *y*, *z*, *x*; (v) *z*, *x*, *y*; (vi) −*y*+1/2, −*z*, −*x*+1/2; (vii) −*y*, −*z*+1/2, −*x*+1/2; (viii) −*z*+1/2, −*x*+1/2, −*y*; (ix) −*z*, −*x*+1/2, −*y*+1/2; (x) −*x*+1/2, −*y*+1/2, −*z*; (xi) −*x*+1/2, −*y*, −*z*+1/2; (xii) −*y*, −*z*, −*x*; (xiii) −*z*, −*x*, −*y*; (xiv) −*x*, −*y*, −*z*; (xv) −*y*+1/2, −*z*+1/2, −*x*+1; (xvi) −*z*+1/2, −*x*+1, −*y*+1/2; (xvii) −*x*+1, −*y*+1/2, −*z*+1/2; (xviii) *x*, *y*−1/2, *z*−1/2.
